# Atomistic Insights into Aluminum Doping Effect on Surface Roughness of Deposited Ultra-Thin Silver Films

**DOI:** 10.3390/nano11010158

**Published:** 2021-01-10

**Authors:** Zhong Tian, Han Yan, Qing Peng, Lin Jay Guo, Shengjun Zhou, Can Ding, Peng Li, Qi Luo

**Affiliations:** 1 School of Mechanical and Electronic Engineering, Wuhan University of Technology, Wuhan 430070, China; tianzhong@whut.edu.cn (Z.T.); dcinwhut@whut.edu.cn (C.D.); lp1968@whut.edu.cn (P.L.); 2 Physics Department, King Fahd University of Petroleum & Minerals, Dhahran 31261, Saudi Arabia; 3 K.A.CARE Energy Research & Innovation Center at Dhahran, Dhahran 31261, Saudi Arabia; 4 Department of Electrical Engineering and Computer Science, University of Michigan, Ann Arbor, MI 48109, USA; guo@umich.edu; 5 School of Power and Mechanical Engineering, Wuhan University, Wuhan 430072, China; zhousj@whu.edu.cn; 6 School of Sciences, Wuhan University of Technology, Wuhan 430070, China; luoqi871026@gmail.com

**Keywords:** molecular dynamics, surface morphology, ultra-thin silver film

## Abstract

Ultra-thin and continuous metallic silver films are attracting growing interest due to the applications in flexible transparent conducting electrodes. The surface morphology and structure of silver film are very important for its electrical resistivity and optical loss. Therefore, roughness control is essential for the production of ultra-thin metallic electrode film. We have investigated the effect of aluminum doping on the improvement of surface morphology of ultra-thin silver films using molecular dynamics simulations. Al-doped silver films showed smaller surface roughness than pure silver films at various substrate temperatures. When the temperature of the substrate was 600 K, the roughness of Al-doped silver film first decreased, and then increased with the increase of the incident velocity of silver atoms. Silver atoms were more likely to agglomerate on the surface of the substrate after adding aluminum atoms, as aluminum dopants promoted the immobilization of silver atoms on SiO_2_ substrate due to the anchoring effect. The smoother surface could be attributable to the reduced mean free path of silver due to the cage effect by the aluminum dopant.

## 1. Introduction

Ultra-thin metal films are wildly applicated in metamaterials, plasmonic devices, nanophotonic, and light-emitting diodes [1,2]. Recently, continuous metallic films became a good candidate for the replacement of indium tin oxide (ITO) for flexible optoelectronic devices [3,4,5]. Silver is considered to be the best material for transparent conducting electrodes among metals, due to its low electrical resistance and low refractive index, as well as its high permeability from visible to near-infrared optical wavelengths [6,7]. The performance of deposition film is closely related to its surface morphology, for instance, it has been proven that surface roughness at the atomic scale greatly affects the electrical conductivity of copper films, due to the destruction of the isotropic Fermi surface [8]. Therefore, as an essential material of transparent electrodes, an ultra-thin and smooth surface is urgently needed. Although large area metal films can be easily prepared by electron beam and magnetron sputtering, rough surface morphology is often obtained during the deposition process, as a result of thin silver films grown in the Vomer–Webber mode on polycrystalline substrates [9,10]. To address this issue, much experimental and theoretical research on the fabrication of smooth ultra-thin silver films has been carried out. Nabiyouni et al. grew silver atoms on gold substrates by electrodeposition, and the results show that the grain size and roughness increase as the substrate rotation speed increases [11]. Ko et al. prepared an ultra-smooth and ultra-thin silver film on Corning glass by using an AlN seeding layer. The results indicate that the AlN seeding reduces the percolation threshold of silver films and formed a smooth surface [12]. Jamnig et al. studied the effect of nitrogen gas surfactant on the growth evolution of nanoscale silver films on silicon dioxide substrates, and the results show that early nitrogen deployment leads to 2D morphology without affecting the resistivity of silver film [13]. Kawamura et al. studied the suppression effect of titanium atoms on the agglomeration of thin silver film, and they found that adding titanium atoms can improve the adhesion of silver film to substrate [14]. Pliatsikas et al. studied the surface morphological evolution of magnetron-sputtered thin silver films deposited on silicon dioxide substrates, and found that silver layers grow flatter in the oxygen-containing gas atmosphere due to incomplete island coalescence. However, they also found that oxygen causes the increase of electrical resistivity of the silver layers [15]. Wang et al. provided an effective method for the fabrication of transparent silver electrodes and found that minimal oxygen-doping significantly improves the optical and electrical performances of silver films [16]. Moreover, Gu et al. investigated the microstructure characterizations and found that Al-doped silver film was smoother than pure silver film [17]. Zhang et al. developed Al-doped silver films on fused silica substrate and investigated the electrical and optical properties, and the results show that organic photovoltaic devices using Al-doped silver films as transparent cathodes produce better efficiency than those made with ITO electrodes [18]. The team also showed that such ultra-thin transparent conductors can benefit the light output in organic light-emitting diode (OLED) devices [19]. Ji et al. demonstrated a flexible transparent electrode consisting of ultra-thin and ultra-smooth copper-doped silver film [20]. High-efficiency transparent organic photovoltaics have recently been realized by using the doped silver film [21]. These results reveal that doping in the deposition process can greatly improve the performance of ultra-thin silver film electrodes. A recent review of the vast amount of experimental investigations on ultra-thin silver films has been presented by Zhang et al. [22]. However, despite many experimental results and characterizations, microscopic studies on how aluminum doping affects the initial deposition processes of silver films have been lacking.

In this paper, molecular dynamics (MD) simulations have been employed to investigate the aluminum doping effect on the deposition and growth process of silver films on amorphous SiO_2_ substrate. Different deposition parameters, including the substrate temperature and incident velocities, were considered. Comparative analysis and a discussion of surface morphology and the initial formation between the pure silver film and the Al-doped silver film were carried out. This paper is expected to provide a theoretical reference for the preparation of high-quality ultra-thin silver film. For example, the kinetic energy of the incident atom is usually achieved by increasing the sputtering current power, whereas the power adjustment affects the concentration of the incident atom. Therefore, a separate discussion of the effects of concentration and incident kinetic energy in atomistic insights would be helpful for further investigation.

## 2. Model and Methods

**Figure 1:**
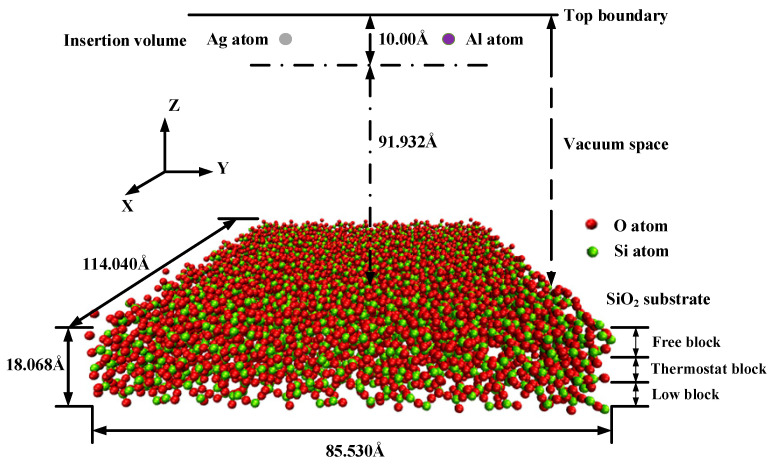
Schematic of simulation model of the deposition process.

Molecular dynamics simulation is a well-established approach that is widely used for computational sub-micron scale investigations [23,24]. The simulations of the silver film deposition process were carried out using the Large-scale Atomic/Molecular Massively Parallel Simulator (LAMMPS) code [25,26]. The simulation box was 114.04 Å (*x*-axis) × 85.53 Å (*y*-axis) × 120.00 Å (*z*-axis) as a super unit cell, which comprised the substrate region and the deposited film region, as shown in Figure 1. Periodic boundary conditions were applied along the X and Y directions of the simulation box. The bottom and top boundaries in the Z direction were non-periodic. The coordinates of the box boundaries were fixed.

The amorphous SiO_2_ substrate was modeled as a tetrahedra-like structure and consisted of 3420 silicon atoms and 6912 oxygen atoms, which was defined as three blocks: the fixed block, the thermostat block, and the free block. The fixed block was defined from the bottom to 5 Å to prevent the substrate from slipping during the deposition along the Z direction, in which the atoms were fixed and could not move out through the bottom boundary. The thickness of the fixed block was proven to be sufficient in deposition simulations by a previous report [27]. The thermostat block was defined from 5 Å to 11 Å to absorb the kinetic energy of the incident atoms during the deposition process. The free block was defined from 11 Å to 18 Å to simulate the interactions and motions. Atoms aggregated in this block after the impacts of the deposited atoms. The atoms present in the thermal control block were free to move, and the initial velocities of them were governed by the Maxwell–Boltzmann distribution [28]. The temperature of the free block was transferred to the thermal control block through heat conduction. A vacuum layer of about 90 Å was arranged above the substrate, in which the deposition atoms of silver and aluminum were injected from the top of the vacuum space. The equations of the motions of the atoms were computed by the Verlet time integration algorithm, and the simulation time step was set to 2 fs. Different incident velocities and temperatures that affected the deposition of aluminum atoms and silver atoms on the SiO_2_ were considered.

The parameters of potential functions and corresponding interactions between atoms are very important for the accurate prediction of the dynamics and material properties. The parameter set for the Tersoff potential [29,30] had been used to study the structural properties of the Si-O system, which is an empirical function composed of two-body terms depending on the direct situations. Interactions between the silver and the silver atoms were modeled using the embedded-atom method (EAM) potential, which is applicable to metallic atom systems due to combining pair interactions with the atomic embedding energy term depending on the local electron density [31]. The spherically symmetric Lennard–Jones (LJ) potential was used to represent the dynamic interactions between the atoms. The Lennard–Jones potential E_LJ_ is defined below:

(1) $$E^{LJ}\left(\gamma_{ij}\right)=4\varepsilon \left[{\left(\frac{\sigma }{\gamma_{ij}}\right)}^{12}-{\left(\frac{\sigma }{\gamma_{ij}}\right)}^{6}\right]$$

In the formula above, γ*_ij_* is the distance between atom *i* and atom *j*; ɛ is the height of Lennard–Jones potential energy. When Lennard–Jones potential energy is used for mixed materials, the two elements (ɛ*_ij_*, σ*_ij_*) should be using the Lorentz–Berthelot combination rules [32]. The *LJ* parameters are listed in Table 1.

The open visualization tool (OVITO) was used for the visualization of the calculation results, displaying the deposition process and the reactions to surface morphology [36]. The surface roughness of the deposited films was presented by the root-mean-square (RMS) R_s_, which was computed as follows [37]:

(2) $$R_{s}=\sqrt{\frac{\sum_{i=1}^{n}{\left(Z_{i}-Z_{\mathrm{mean}}\right)}^{2}}{n}}$$

where Z_i_ is the Z coordinate of the topmost atoms in each region, Z_mean_ is the mean height of the Z coordinate of the topmost atoms in each region, and n is the total number of regions divided. In this quantitative evaluation, the deposition surface was divided into 55 × 42 regions along the X-Y plane.

## 3. Results and Discussion

### 3.1. Effects of Aluminum Composition

**Figure 2:**
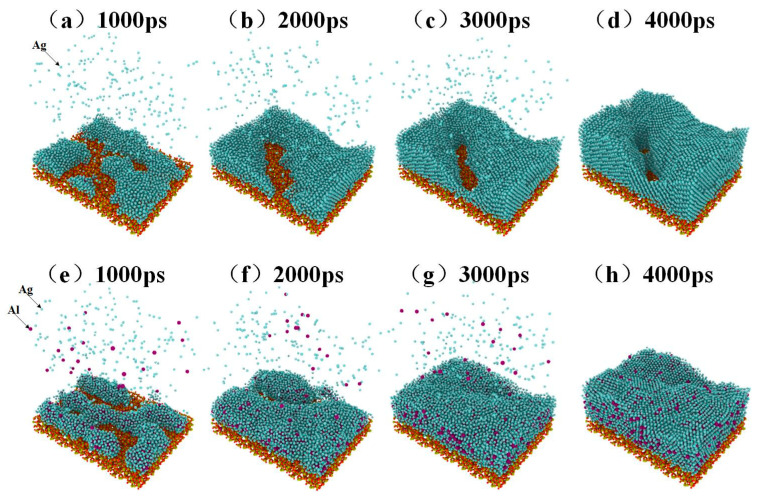
The growth process of pure silver film from (**a**) to (**d**), and the growth process of Al-doped silver film from (**e**) to (**h**).

The deposition process of pure silver film and Al-doped silver film on SiO_2_ substrate are shown in Figure 2. In this process, a total of 22,000 atoms were deposited. The pure silver film contained 22,000 silver atoms, while the Al-doped silver film contained 20,000 silver atoms and 2000 aluminum atoms. The temperature of the substrate was maintained at 600 K. The initial incident velocity of the silver and aluminum atoms was 2 Å/ps, and the incident angle of each deposited atom is perpendicular to the substrate surface.

Four snapshots of different times are presented to describe the deposition process. Figure 2a–d shows the growth process of pure silver film, while Figure 2e–h shows the growth of Al-doped silver film. At the initial deposition stage, there were many clusters, as shown in Figure 2a,e. As the deposition continues, the clusters are connected with each other. Pure silver deposition forms island-like structures, as shown in Figure 2c. Compared to pure silver deposition, Al-doped silver deposition forms relatively smooth surfaces, as shown in Figure 2f. At the final deposition stage, there were still some island-like structures in the pure silver deposition, as shown in Figure 2d, which presents a rough and discontinuous surface morphology. The Al-doped silver deposition formed a smooth and continuous surface morphology, shown in Figure 2h. These results suggest that adding a small amount of aluminum atoms is helpful for forming a smooth and continuous silver film.

**Figure 3:**
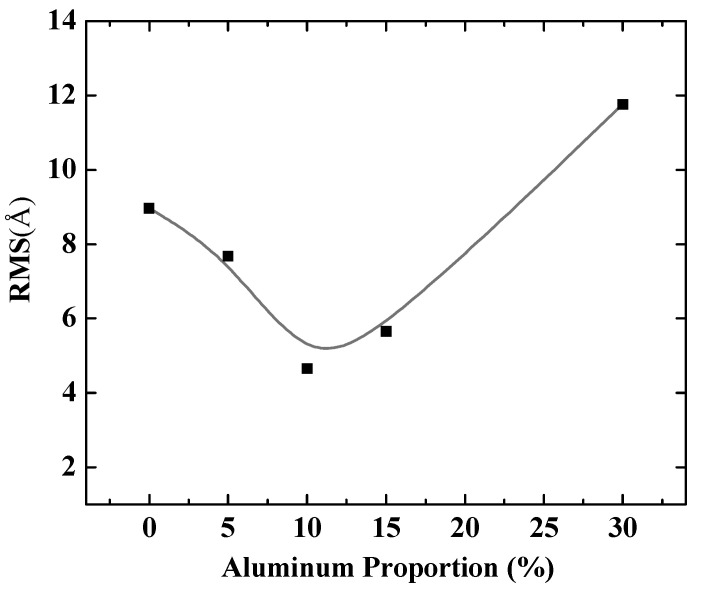
The RMS of Al-doped silver films versus proportion of aluminum in deposited atoms.

To further quantify the effects of aluminum composition on Al-doped silver film, different doping ratios of aluminum in silver films were considered. Figure 3 shows the RMS of Al-doped silver films on SiO_2_ substrates, with the aluminum proportion ranging from 0% to 30% in the deposited atoms. It is obvious that adding aluminum atoms greatly reduced the roughness of the film surface compared to pure silver film (the aluminum proportion was 0%). Moreover, the surface morphology of Al-doped silver films gradually became rough when the proportion of aluminum atoms was above 10%. That is, the best doping concentration ratio for Al-doped silver film was 10% at a temperature of 600 K, while the value of RMS was around 4.65 Å.

### 3.2. Effect of Substrate Temperature

The temperature of the substrate is also a key parameter for the deposition. In this section, the effects of the deposition temperature of the substrate on the surface morphology of ultra-thin silver film were simulated and discussed. For pure silver deposition cases, 22,000 silver atoms were deposited in each calculation, while 20,000 silver atoms and 2000 aluminum atoms were simultaneously deposited in each calculation of Al-doped deposition. The temperatures of the substrate were increased from 450 K to 900 K.

**Figure 4:**
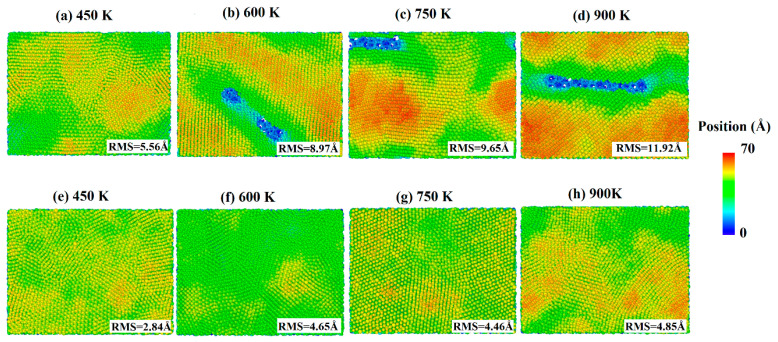
Surface morphology of pure silver films (**a**–**d**) and Al-doped silver films (**e**–**h**), in which the temperature of the substrate increased from 450 K to 900 K.

The snapshots of the deposited process of pure silver film and Al-doped silver film on SiO_2_ substrate are shown in Figure 4, where the atoms are colored by the height of the *z*-axis. As shown in Figure 4a–e, the surfaces of the pure silver film were rougher than those of the Al-doped ones at all temperatures. As the temperature increased, the island-like structures in silver films became more significant. This is likely due to the increased atomic mobility on the surface, which increases the probability of silver atom aggregation and clustering. Experimental studies have also shown that the effects of growth temperatures on morphology are affected by the substrate surface [38]. The surfaces of the Al-doped silver films were smoother and more continuous than the pure silver films at different temperatures, as shown in Figure 4f–h. Recently, kinetic Monte Carlo (kMC) simulations of silver depositions on weakly-interacting substrates showed that higher temperatures promote top-layer nucleation, resulting in an increase in island height-to-radius aspect ratios [39].

**Figure 5:**
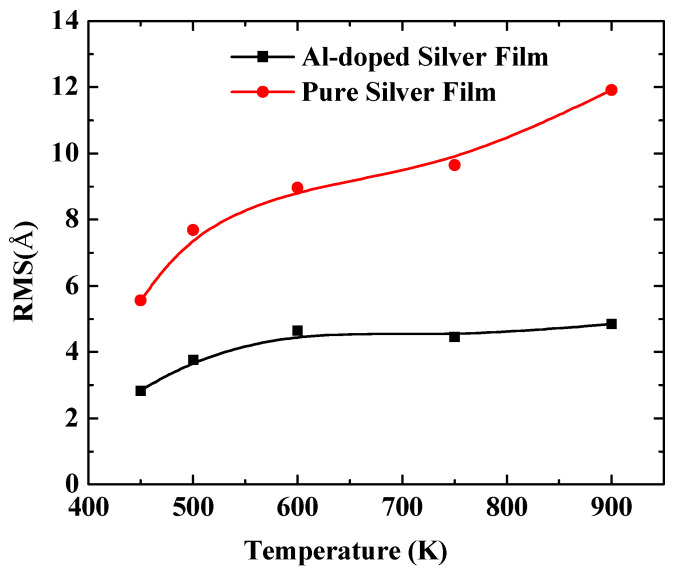
The RMS surface roughness of pure silver films and Al-doped silver films versus substrate temperatures.

In order to further quantify the effects of substrate temperatures on the surface morphology, the RMS of silver films were calculated by a statistical atom coordinate. The calculation results are presented in Figure 5, where the red curve denotes the pure silver film, and the black curve denotes the Al-doped silver film. The RMS increased as the substrate temperature increased from 450 K to 900 K. The slope of the red curve is larger than that of the black curve, which means the Al-doped silver film was more thermally stable than the pure silver film as the temperature increased. The RMS of the pure silver film reached 11.92 Å when the substrate temperature reached 900 K, while the RMS of the Al-doped silver film tended to be stable at around 4.85 Å at that temperature. Additive aluminum atoms benefited the thermal stability of the formed film in the deposition of the silver atoms. The evolutions of the top-layer critical radius (Rc) and the growth temperature were affected by a proportionality constant α. In the case of a high α, the Rc increased as the growth temperature increased, while a small α case showed a decreasing Rc as the growth temperature increased [39]. Therefore, it is possible that the doping of aluminum affects the value of this proportionality constant.

### 3.3. Effects of Initial Incident Velocity of Deposition Atoms

**Figure 6:**
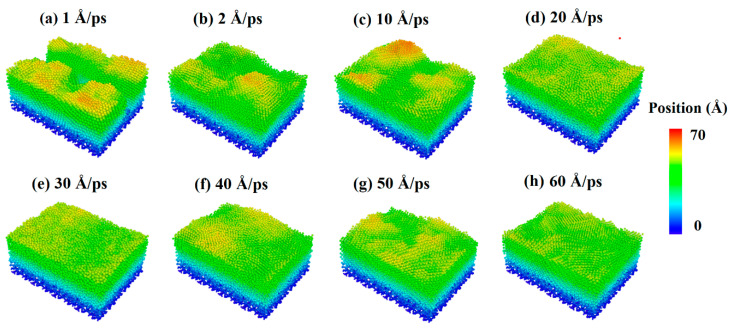
Surface morphology of Al-doped silver films deposited under different incident velocities of the aluminum atoms at 600 K.

The source power of sputtering or e-beam is also a key parameter for the fabrication of ultra-thin silver film electrodes, which determines the initial kinetic energy of deposition atoms. Therefore, the parameter of the incident velocity of silver and aluminum atoms was introduced into the study of Al-doped silver film deposition. In order to analyze the influence of aluminum atomic velocity independently, the incident velocity of aluminum atoms changed from 1 Å/ps to 60 Å/ps, and the incident angle from the *z*-axis was set to 0 degrees, while the incident velocity of the silver atoms was maintained at 2 Å/ps. In each calculation, 20,000 silver atoms and 2000 aluminum atoms were deposited. The temperature of the substrate was set to 600 K in each simulation case. The surface morphologies of the Al-doped silver films grown on SiO_2_ substrate at different incident velocities of aluminum atoms are shown in Figure 6, where the atoms are colored by the height of the *z*-axis. The surface morphology of the Al-doped silver film became smoother as the incident velocity of the aluminum atoms increased.

**Figure 7:**
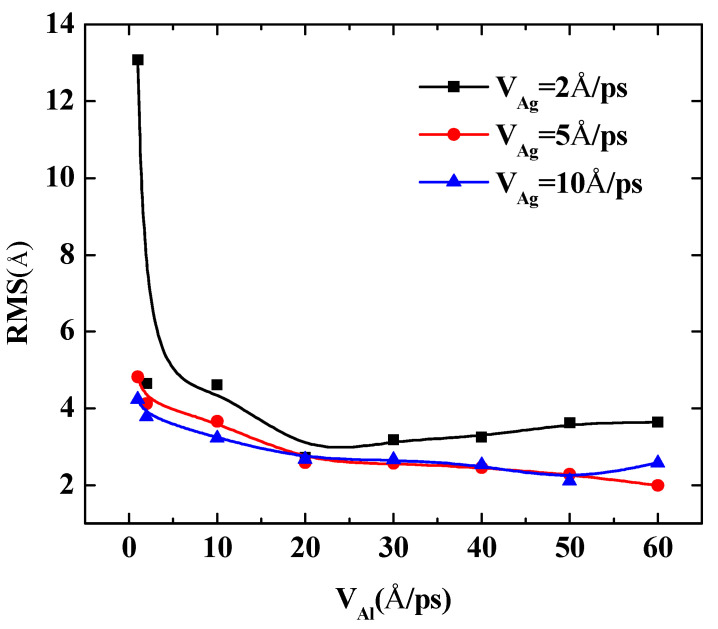
The RMS surface roughness of Al-doped silver films deposited in various initial incident velocities of aluminum atoms at 600 K.

To further quantify the effects of the initial incident velocity of aluminum atoms on the surface roughness of Al-doped silver film with different incident velocities of silver atoms, two more cases were calculated, in which the incident velocities of silver atoms were set at 5 Å/ps and 10 Å/ps, respectively. The RMS results of the Al-doped silver film versus the initial incident velocity of the aluminum atoms are presented in Figure 7.

The surface roughness of Al-doped silver films significantly decreased as the velocity of the aluminum atoms increased from 1 Å/ps to 10 Å/ps. In the case of the velocities of the silver atoms being 5 Å/ps and 10 Å/ps, as the value of velocity continued to increase, the surface roughness of the Al-doped silver films decreased slightly. On the contrary, in the case of the velocity of the silver atoms being 2 Å/ps, the surface roughness of the Al-doped silver films increased slightly as the value of velocity continued to increase. These results indicate that a higher initial kinetic energy of aluminum atoms is beneficial to the formation of better surface morphology. Similar experimental research on sputtered silver films revealed that silver film prepared at plasma power 25 W contained more discontinuous pores than the film prepared at plasma power 50 W, and the surfaces are smoother when the plasma power increased to 75 W and 100 W [40]. Another similar result has been reported by Gu et al. [17]. However, it was found that the RMS of Al-doped silver films increase again as the incident velocity of aluminum atoms exceeds 50 Å/ps, in the case of the velocity of silver atoms being 5 Å/ps, which means that the incident energy of silver atoms should be controlled in a reasonable range within 1 to 50 Å/ps.

**Figure 8:**
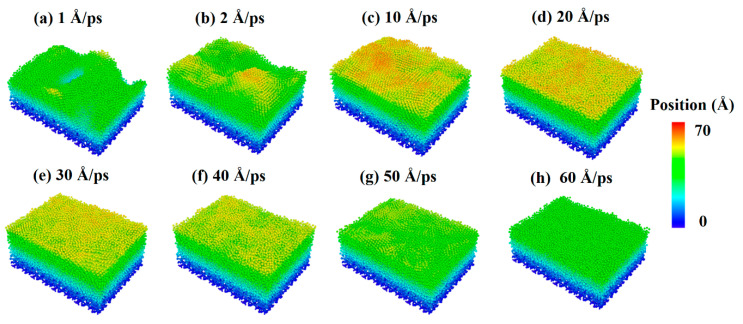
Surface morphology of Al-doped silver films on SiO_2_ substrate deposited in different incident velocities of silver atoms at 600 K.

The incident velocity of silver atoms should be discussed further to explore the effect of the incident velocity of deposition atoms on the surface morphology of Al-doped silver films. Figure 8 shows the surface morphology of Al-doped silver films deposited on SiO_2_ substrates at different incident velocities of silver atoms. The incident velocity of aluminum atoms was 2 Å/ps. The atoms are colored by the height of their coordinate along the *z*-axis. It is obvious that the surface morphology of Al-doped silver films was rough at 600 K, while it became smooth as the incident velocity of the silver atoms increased. This is because the high kinetic energy of aluminum atoms can overcome the random thermal motion of the substrate atoms and form a chemical bond with oxygen.

**Figure 9:**
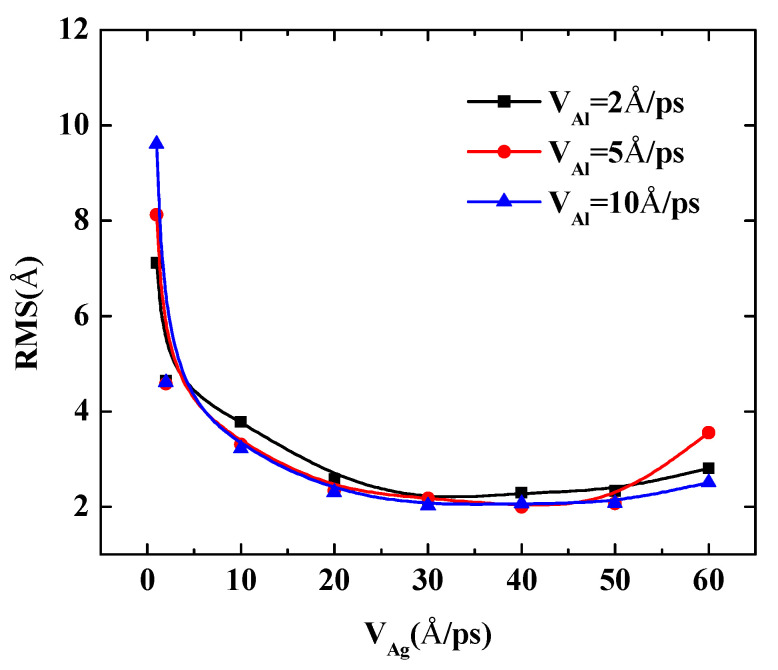
The RMS surface roughness of Al-doped silver films deposited in different incident velocities of silver atoms at 600 K.

The following quantity calculations present the deposition process of Al-doped silver films that the incident velocity of silver atoms varies from 1 Å/ps to 60 Å/ps are shown in Figure 9. Three doping conditions were considered, in which the incident velocities of aluminum atoms were set at 2 Å/ps, 5 Å/ps, and 10 Å/ps, respectively. The temperature of the substrate was also maintained at 600 K in each calculation case. The RMS of the Al-doped silver films were reduced rapidly when the initial incident velocity of the silver atom was less than 30 Å/ps. However, the reduction of the RMS of Al-doped silver film became stable when the range of incident velocities of silver atoms was between 30 Å/ps and 50 Å/ps. When the incident velocity of deposited silver atoms exceeded 50 Å/ps, the kinetic energy of silver atoms was more than 14 eV, and the surface morphology of the Al-doped silver film turned rough. This may partially be due to the fact that the incident silver atoms passed through the surface of the silver film that was formed before, and directly injected into the lattice of silver film. The RMS of the Al-doped silver film reached a minimum of 2.03 Å, in the case of the velocity of the aluminum atoms being 10 Å/ps, and the velocity of the silver atoms increased to 30 Å/ps. Thus, the surface morphology was not solely decreasing with the increasing incident velocity of the deposition atoms, but decreased rapidly at first and then increased slightly.

### 3.4. Atomic Migration Mechanism

**Figure 10:**
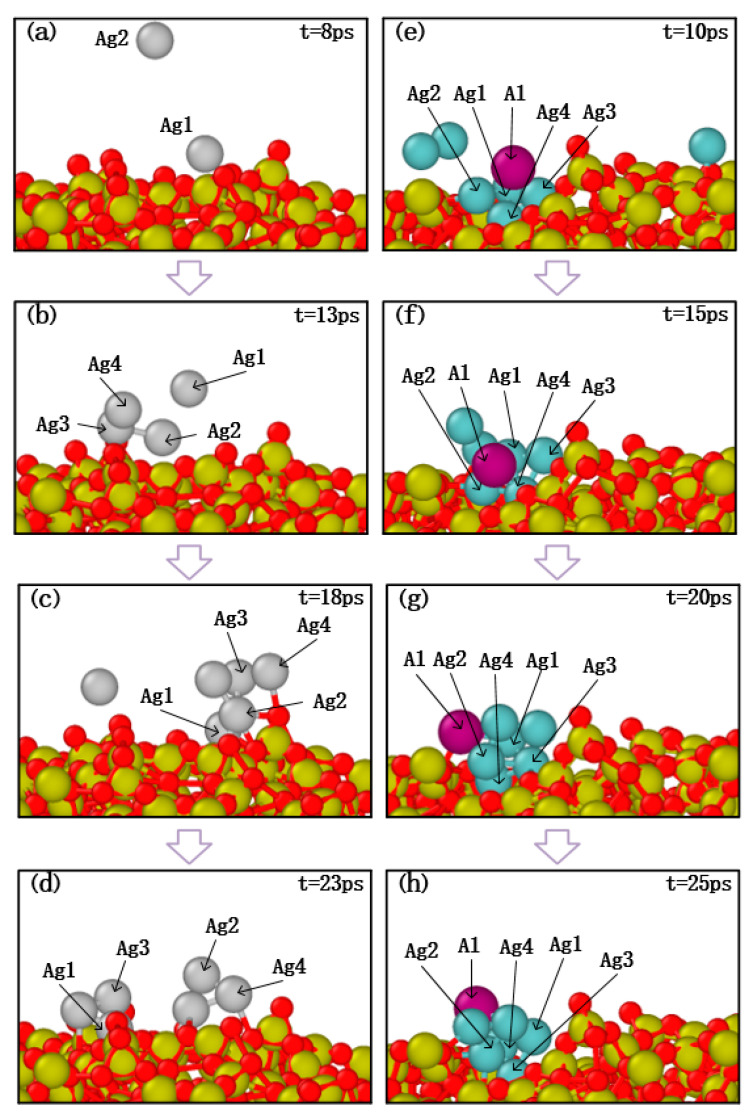
Atomic migration process of pure silver atom deposition cases (**a**–**d**) and aluminum doping cases (**e**–**h**).

Taking the above results into consideration, the morphological evolutions of ultra-thin Al-doped silver film revealed that a few aluminum atoms may strongly influence the migration and subsequent growth of silver films. Further research is presented to discuss the effects of aluminum atom doping on the initial deposition stage. The migration characteristics of silver atoms and aluminum atoms on SiO_2_ substrate are shown in Figure 10, in which the growth temperature was set to 600 K and the proportion of injected atoms was set as Ag:Al = 10:1. As can be seen in Figure 10a,b, silver atoms were successfully deposited on the surface and then bonded with the appropriate atoms. Subsequently, as the number of deposited silver atoms increased, it was found that the silver atoms dispersed. This may be due to the weak interaction between the silver atoms and the SiO_2_ substrate.

In the deposition of the aluminum doping case, several silver atoms with a single aluminum atom were deposited on the substrate, and the snapshot results are shown in Figure 10e,f. The deposition results show that the silver clusters with an aluminum atom still attached together as the number of silver atoms increased. Therefore, these processes indicate that silver atoms are more likely to agglomerate on the surface of the substrate after adding aluminum atoms, and aluminum atoms mitigate the migration behavior of silver atoms, which confirms that the smoothness of Al-doped silver film is better than pure silver film.

Furthermore, reports show that the bond strength of Ag-O bonds is much weaker than that of Al-O bonds [41]. This is because of the high enthalpy of formation of Al-O bonds. In the initial deposition stage, the average diffusion distance of silver atoms on SiO_2_ substrate is larger than that of aluminum atoms, and aluminum atoms are more easily attached to SiO_2_ substrate than silver atoms due to their stronger bond with the oxidized surface. Then, the immobilization of the aluminum atoms promotes the immobilization of the silver atoms on the SiO_2_ substrate. It can be explained by the anchoring effect [42,43], in which the aluminum atoms anchored to the substrate act as a nucleation center to a silver cluster. A further explanation is that when silver atoms try to move some distance from the aluminum atoms, the cluster will become smaller and their surface areas and surface energies increase, therefore, energetically, it is not favorable.

The reduced roughness due to the additive aluminum atoms in the subsequent growth process could be further explained by the sluggish migration, according to the cage effect [44], in which aluminum doping atoms act as obstacles for the movement of silver clusters in bulk diffusion. Similar analysis has been found in the experiment of germanium-wetted silver films, in which the Raman spectra results demonstrated that the germanium atoms at the silver grain boundaries form clusters of a few atoms [45]. Because of the mismatch of the atom size, additive aluminum atoms create atomic distortions and strains. Such a strain field is centered on these impurities and is long-range. As a result, these additive heterogeneous aluminum atoms form cages that impair the migration of silver atoms by reducing the mean free path. According to the cage model [46], the mean free path *λ* is a function of the concentration *c* of impurity, as λ=A/∛c+B, where *A* and *B* are constants. Increases in the concentration of aluminum results in the reduction of the mean free path of silver, and thus, the agglomerations. As a consequence, the roughness decreases.

## 4. Conclusions

We have systematically studied the effects of aluminum doping on the initial growth of ultra-thin silver films using molecular dynamics simulations. The critical conditions of Al-doped silver films under different aluminum compositions, substrate temperatures, and initial incident velocities were calculated, and the surface morphology and RMS surface roughness of deposited film were discussed. The results show that aluminum dopant promoted the immobilization of silver atoms on SiO_2_ substrate and formed a smoother silver film. Further studies indicated that Al-doped silver films show better surface roughness than pure silver films at various substrate temperatures. Moreover, when the temperature of the substrate is set to 600 K, the RMS of the Al-doped silver film first decreased, and then increased with the increasing of the incident velocity of deposited silver atoms. The optimum injection velocity was 30 Å/ps for the silver atoms and 10 Å/ps for the aluminum atoms. The atomic migration mechanism is that silver atoms are more likely to agglomerate on the surface of the substrate after adding aluminum atoms, as aluminum dopants promote the immobilization of silver atoms on SiO_2_ substrate, due to the anchoring effect. The smoother surface could be attributable to the reduced mean free path of silver, due to the cage effect by the aluminum dopant. The simulation results of this paper could be beneficial in guiding and regulating the deposition process and analyzing the film characteristics of transparent metallic conducting electrode films.

## Figures and Tables

**Table 1:** *LJ* parameters and material constants [33,34,35].

Atom	Ε (eV)	σ (Å)
Ag-Ag	0.345	2.644
Al-Al	0.392	2.620
Si-Si	0.0175	3.826
O-O	0.0026	3.166
Al-O	0.032	2.893
Ag-O	0.030	2.905
Al-Si	0.083	3.223
Ag-Si	0.078	3.235

## Data Availability

The data presented in this study are available on request from the corresponding author H.Y.
